# The island–mainland species turnover relationship

**DOI:** 10.1098/rspb.2012.0816

**Published:** 2012-08-08

**Authors:** Yoel E. Stuart, Jonathan B. Losos, Adam C. Algar

**Affiliations:** 1 Museum of Comparative Zoology and Department of Organismic and Evolutionary Biology, Harvard University, 26 Oxford Street, Cambridge, MA 02138, USA; 2 School of Geography, University of Nottingham, Sir Clive Granger Building, Nottingham NG7 2RD, UK

**Keywords:** beta diversity, environmental dissimilarity, geographical distance, neotropics, species richness

## Abstract

Many oceanic islands are notable for their high endemism, suggesting that islands may promote unique assembly processes. However, mainland assemblages sometimes harbour comparable levels of endemism, suggesting that island biotas may not be as unique as is often assumed. Here, we test the uniqueness of island biotic assembly by comparing the rate of species turnover among islands and the mainland, after accounting for distance decay and environmental gradients. We modelled species turnover as a function of geographical and environmental distance for mainland (M–M) communities of *Anolis* lizards and Terrarana frogs, two clades that have diversified extensively on Caribbean islands and the mainland Neotropics. We compared mainland–island (M–I) and island–island (I–I) species turnover with predictions of the M–M model. If island assembly is not unique, then the M–M model should successfully predict M–I and I–I turnover, given geographical and environmental distance. We found that M–I turnover and, to a lesser extent, I–I turnover were significantly higher than predicted for both clades. Thus, in the first quantitative comparison of mainland–island species turnover, we confirm the long-held but untested assumption that island assemblages accumulate biodiversity differently than their mainland counterparts.

## 1. Introduction

Oceanic islands and archipelagos are often characterized by high rates of endemism [1] that probably result from rapid speciation (anagenetic and cladogenetic) on islands of sufficient area and isolation [2–7]. For example, the Hawaiian Archipelago hosts several classic adaptive radiations, including silverswords and honeycreepers. The oft-cited pattern of high island endemism suggests that a unique combination of processes may govern assembly on islands. However, many mainland areas also house endemic biotas with endemism levels that can rival the classic adaptive radiations of oceanic islands [8], especially in mountainous habitat islands characterized by dispersal barriers and steep environmental gradients [9]. Even in less mountainous regions, differences in habitat type, climatic gradients or spatial separation can lead to substantial turnover across space [10,11]. The existence of mainland communities that harbour similar levels of endemism to islands suggests that island and mainland assembly may be more similar than currently recognized and that islands may not be the unique generators of diversity they have long been assumed to be.

Here, we focus on species turnover along geographical and environmental gradients to test for an island effect on biotic assembly. Mainland–island species diversity relationships have been, and remain, a stimulus of evolutionary and biogeographic theory [7,12–14]. However, the predominant focus has been on the species richness of individual islands relative to the mainland. In contrast, the mainland–island relationship for species turnover remains undescribed and unexplored. To remedy this, we compare rates of species turnover within the mainland (M–M) with rates of mainland–island (M–I) and island–island (I–I) turnover for two species-rich herpetofaunal radiations in the Caribbean and Neotropics: *Anolis* lizards and Terrarana frogs.

The rate of species turnover has generally been modelled as a function of geographical distance and environmental dissimilarity [10,11,15]. Large geographical distances may generate high species turnover by lowering the probability of species exchange through dispersal and increasing the probability of anagenetic speciation through reduced gene flow, while environmental dissimilarity can lead to high turnover as a result of environmental filtering or ecological speciation during local adaptation to different environments. Thus, high species turnover between islands or between islands and the mainland is not necessarily indicative of unique island assembly; M–I and I–I species turnover may be high but still consistent with M–M turnover for a given geographical isolation and environmental dissimilarity.

Alternatively, there may be an added ‘island effect’ on species turnover beyond that expected from geographical distance and environmental dissimilarity that stems from the inhospitable overwater dispersal barrier surrounding islands; comparatively reduced dispersal, limited gene flow and increased ecological opportunity on islands may drive unique assembly of island floras and faunas. In this case, the M–M model would poorly predict M–I and I–I species turnover. To our knowledge, this island effect has never been quantitatively tested.

## 2. Methods

### (a) Study organisms

*Anolis* lizards (Iguanidae) and Terrarana frogs (*sensu* [16]; Leptodactylidae) have radiated extensively in the Caribbean and New World tropics, with approximately 400 and 850 species, respectively. Both clades are insectivorous, include species that are arboreal, terrestrial or partially aquatic [16,17], and lay direct-developing eggs [17,18]. Phylogenetic and biogeographic reconstructions suggest that both clades originated in the mainland Neotropics, colonized the Caribbean islands and back-colonized the mainland from the Caribbean once, where they radiated again [19,20].

### (b) Mainland and Caribbean faunas

#### (i) Species lists

We defined terraranans according to two recent studies [16,20] and anoles following Algar & Losos [21]. We built Caribbean species lists for both clades in November 2011 by cross-referencing published lists [16,17,20,22] against the online databases CaribHerp (www.caribherp.org), HerpNET (www.herpnet.org) and Amphibian Species of the World 5.5 (http://research.amnh.org/vz/herpetology/amphibia). For mainland terraranans, we considered the 865 terraranan species with IUCN range maps (www.iucnredlist.org/technical-documents/spatial-data), 714 of which were mainland species. For mainland anoles, we used the 203 species range maps built by Algar & Losos [21].

#### (ii) Island species composition

Using the resources mentioned earlier, we determined the anole and terraranan species composition of Caribbean islands for which we could obtain environmental data, resulting in 65 islands for anoles and 46 islands for terraranans (19 islands that contained anoles did not contain terraranans; electronic supplementary material, table S1). For comparison, we also extracted terraranan island species composition from the IUCN range maps (there are no island IUCN range maps for anoles). Species presences that are likely to have resulted from human introductions were excluded.

#### (iii) Mainland species composition

**Figure 1.**
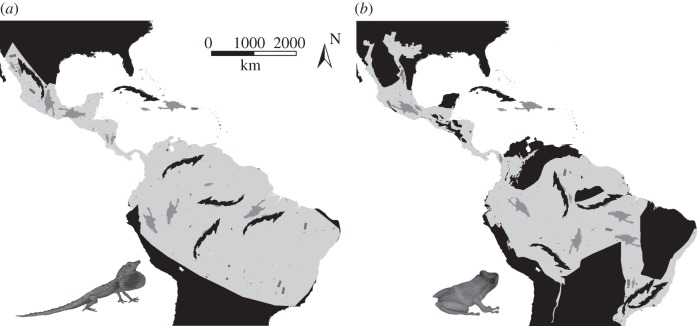
Island-shaped cookie-cutter sampling scheme for (*a*) anoles and (*b*) terraranans. Each island is represented five times on the mainland. The large, light-grey mainland areas depict the region where at least one species is present. Randomly sampled mainland areas are depicted within this region.

We used an island-shaped cookie-cutter approach to define mainland subregions (MSRs) within which species composition could be determined from the IUCN range maps (figure 1; electronic supplementary material, figure S1). Each cookie-cutter's orientation and placement was randomly determined. Owing to the larger land area in South America, purely random placement could result in underrepresentation of more northern environments. To account for this, we first selected a random latitude. Longitude was then randomly chosen from the available land at the chosen latitude. This approach is similar to the spreading-dye method of Algar & Losos [21] except that it preserved island shape as well as size. Each island in our study was represented by five MSRs, resulting in 230 non-overlapping MSRs for terraranans and 325 for anoles. Sampling was performed separately for anoles and terraranans and was limited to regions with at least one anole (or terraranan) species. Following Algar & Losos [21], we excluded the disjunct distribution of *Anolis carolinensis* in the southeast US, as this region was colonized from the Caribbean [23], and whether it should be treated as part of the mainland or as a biogeographic island is unclear.

### (c) Quantifying species turnover

We quantified species turnover between all island and MSR combinations using the Sørensen dissimilarity index, where zero indicates that two sites share the same species assemblage and one indicates no shared species. Turnover measured using Jaccard's index was nearly identical (Pearson's *r* > 0.98 for both clades). Sørensen and Jaccard's indices are commonly used and easily interpretable measures of species turnover. For islands, turnover from the IUCN ranges and from our manually assembled species lists were essentially indistinguishable (Pearson's *r* > 0.99), so only the latter was used as it included two additional islands.

### (d) Quantifying geographical, environmental and area dissimilarity

We measured geographical distance as the minimum straight-line distance among islands and MSRs. The straight-line distance between MSRs was highly correlated with minimum overland distance (Pearson's *r* = 0.99), so we used straight-line distance to maintain consistency with island comparisons. We measured environmental distance between two sampling areas using the Euclidean distance. Environment was quantified using the first five principal components from a principal component analysis on 16 environmental variables derived from the Worldclim dataset (electronic supplementary material, table S2) [24] and mean net primary productivity from the MODIS satellite (productivity data from 2000 to 2010; https://lpdaac.usgs.gov/products/modis_products_table). The environmental variables quantified mean and extreme environmental conditions, as well as spatial and seasonal variation in temperature, precipitation and elevation. The five principal components accounted for 90 per cent (terraranans) and 89 per cent (anoles) of the environmental variation among islands and MSRs. We also calculated all pair-wise differences in area among MSRs and islands. Species turnover and geographical, environmental and area dissimilarity data can be found in the Dryad depository: http://dx.doi.org/10.5061/dryad.gm2p8.

### (e) Model selection and prediction

Treating anoles and terraranans separately, we used multiple regression on distance matrices [25] to determine the within-mainland (M–M) relationship between species turnover and geographical distance, environmental distance and area difference. Because our turnover data were constrained between zero and one and were dominated by these values, we fitted generalized linear models using a logit link. Following Algar & Losos [21] and Algar *et al*. [26], we tested all possible models to identify the best fitting model(s), considering linear, quadratic and all first-order interaction terms as potential predictors. We used Occam's Window [27] based on the Bayesian Information Criterion (BIC) to identify the best set of near-equivalent models for prediction. We evaluated model significance by randomly permuting island and MSR identities 1000 times, and comparing observed BIC and deviance explained (*D*^2^) with this null distribution.

We used Bayesian Model Averaging [27] using the best M–M model(s) chosen by model selection to predict species turnover between MSRs and islands (M–I), and between islands (I–I). Treating M–I and I–I separately, we evaluated predictive performance of the M–M models by regressing observed turnover on predicted turnover [28]. We generated a null distribution of 1000 slope and intercept coefficients by randomly permuting island and MSR identities, re-calculating turnover for M–I (or I–I), and refitting the observed versus predicted regression. If the M–M model poorly predicts the M–I (or I–I) species turnover relationship, then the observed regression coefficients will differ from the null expectation. Importantly, the range of environmental and geographical distances for M–I and I–I comparisons fell within the range of M–M comparisons (electronic supplementary material, figure S2), ensuring that we were not extrapolating beyond the range of the M–M data to predict M–I and I–I patterns.

## 3. Results

### (a) Mainland–mainland relationships

**Figure 2.**
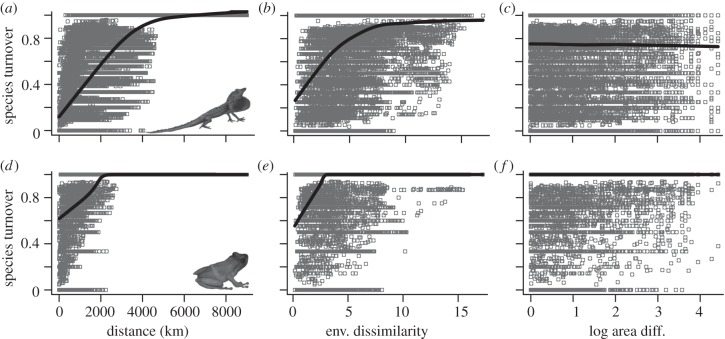
The relationship between species turnover (Sørensen dissimilarity) and (*a,d*) geographical distance, (*b,e*) environmental dissimilarity and (*c,f*) area difference between mainland species assemblages for (*a–c*) anoles and (*d–f*) terraranans. Lines are Lowess curves.

For terraranans, all-subsets regression using BIC and Occam's Window identified a single best model that explained 65 per cent of the deviance:
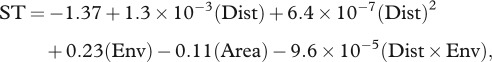
where ST is species turnover, Dist is the geographical distance, Env is the environmental distance and Area is the area difference between MSR sampling units. Turnover was more closely related to geographical and environmental distance than to area difference (figure 2). Observed model BIC and deviance explained were significant according to the permutation test (*D*^2^ = 0.65, *p* < 0.001; BIC = 7092.0, *p* < 0.001).

For anoles, the relationship was similar to that for terraranans (figure 2). However, two models fitted the data almost equally well. One of these models included the same predictors as the best terraranan model with a similar fit (*D*^2^ = 0.62, BIC = 36 466.9):
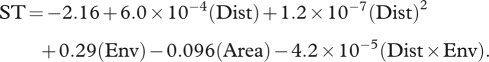


The second model included an additional interaction term between Dist and Area that slightly improved model fit (*D*^2^ = 0.62, BIC = 36 462.5):
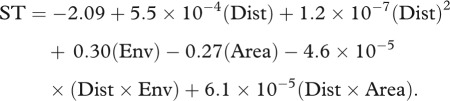


Observed BIC and deviance explained were significant for both anole models according to the permutation test (*p* < 0.001 for both *D*^2^ and BIC statistics).

### (b) Predicting mainland–island relationships

**Figure 3.**
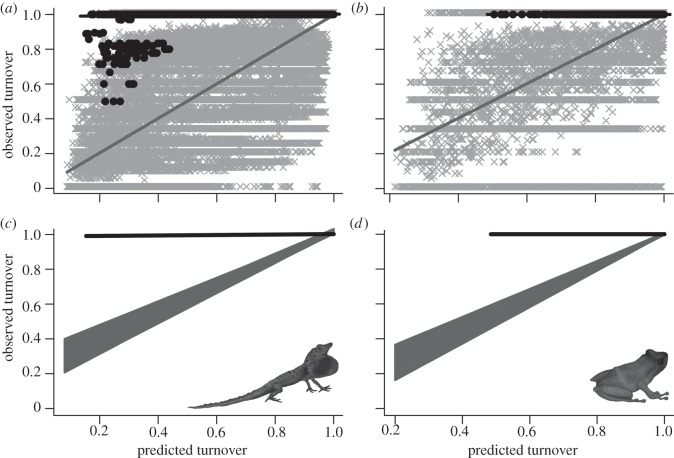
Observed versus predicted species turnover for mainland–island (M–I) comparisons based on the mainland–mainland (M–M) relationship for (*a,c*) anoles and (*b,d*) terraranans. (*a*,*b*) M–M relationships are shown in grey and M–I in black. Note that the abundance of turnover values equal to 1.0 partially obscures the best-fit regression line. (*c*,*d*) The actual M–I regression line (black) relative to 1000 null observed versus predicted relationships (grey).

Species turnover between MSRs and islands was greater than predicted by the M–M model for both anoles and terraranans (figure 3). Slopes from the M–I observed  versus predicted regression were significantly shallower than the null expectation (anoles: 0.013 ± 5.5 × 10^−4^ s.e.; terraranans: 0.0 ± 0.0 s.e.; *p* < 0.001 for both clades), while intercepts were significantly greater than the null expectation (anoles: 0.99 ± 4.4 × 10^−4^; terraranans: 1.0 ± 0.0; *p* < 0.001 for both clades).

### (c) Predicting island–island relationships

**Figure 4.**
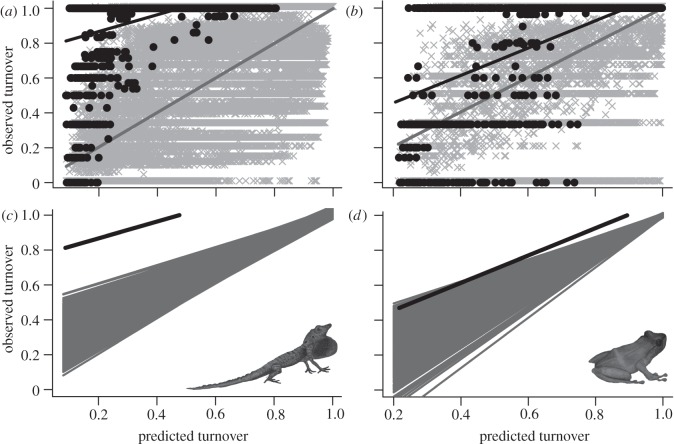
Observed versus predicted species turnover for island–island (I–I) comparisons based on the mainland–mainland (M–M) relationship for (*a,c*) anoles and (*b,d*) terraranans. (*a*,*b*) M–M relationships are shown in grey and I–I in black. (*c*,*d*) The actual I–I regression line (black) relative to 1000 null observed versus predicted relationships (grey).

Patterns of I–I turnover were more accurately predicted than M–I turnover, though accuracy was still low, especially for anoles (figure 4). The *Anolis* slope (0.48 ± 0.03; *p* < 0.001) and intercept (0.77 ± 0.01; *p* < 0.001) from the observed versus predicted I–I regression remained significantly greater and shallower than the null expectation, respectively. For terraranans, the intercept was significantly greater than expected (0.30 ± 0.02; *p* < 0.03), but the slope did not differ significantly from null expectation (0.79 ± 0.03; *p* > 0.10).

## 4. Discussion

### (a) Mainland–mainland species turnover

*Anolis* and Terrarana species turnover among MSRs increased with geographical and environmental distance between assemblages (figure 2), a pattern consistent with turnover patterns of vertebrate and invertebrate taxa worldwide [10,11,29]. The best mainland model for terraranans shared the same predictors and interaction terms as one of the two best models for anoles, suggesting that the two clades accumulate biodiversity in similar ways along spatial and environmental gradients.

Mainland anole assemblages turned over more slowly through geographical and environmental space than terraranan assemblages. Buckley & Jetz [10] linked the rate of species turnover to average range size, with higher turnover in clades composed of smaller-ranged species. Our results are consistent with this pattern; the average range size of terraranans was approximately six times smaller than that of anoles. An average range size–turnover relationship is expected on theoretical grounds [30]; however, rather than a direct causal relationship (e.g. small ranges cause high turnover), turnover pattern differences between anoles and terraranans probably reflect the interaction of dispersal ability, environmental adaptation [10] and biotic interactions, though differences in taxonomic splitting among clades may also play a role (see §4*d*).

### (b) Mainland–island species turnover

M–I species turnover patterns were very similar for both anoles and terraranans (figure 3*a,b*). Species turnover was complete for every terraranan community and nearly every anole community. However, complete or high turnover alone is not sufficient to infer an island effect on species turnover. M–I turnover could still be consistent with the M–M relationship if islands are sufficiently environmentally dissimilar or far away from mainland areas. Our data reject this possibility; for both clades, M–I species turnover was higher than predicted based on the M–M relationship, given M–I geographical and environmental distances (figure 3*c,d*). This island effect on species turnover is probably the result of several related processes. The harshness of the intervening habitat matrix between islands and the mainland (i.e. salt water) probably renders M–I distances greater in terms of dispersal probability when compared with equivalent distances on the mainland. Another key factor is the severe reduction of gene flow from mainland to island populations; without the opposing effects of gene flow [31], island colonizers are more likely to speciate [6]. Finally, ecological opportunity on islands can promote adaptive radiation and diversification [32], leading to high rates of endemism and turnover. These same processes also contribute to differing M–I species richness patterns in anoles [21], and probably in terraranans (Y. Stuart 2011, unpublished data), which may also influence rates of species turnover [33].

### (c) Island–island species turnover

Like M–I species turnover, I–I species turnover was significantly higher for each clade than predicted by the best M–M models (figure 4), again consistent with high speciation (anagenetic and cladogenetic) rates on islands. However, for both clades, the rate at which turnover varied with geographical and environmental distance was closer to the M–M relationship than for M–I comparisons. For terraranans, the slope of the observed versus predicted regression was not significantly different from the null expectation, indicating a similar relationship to that for mainland turnover. Similarly, while the *Anolis* observed versus predicted species turnover regression slope differed significantly from the null expectation, it was closer to the null than M–I turnover (compare panels (*c*) and (*d*) in both figures 3 and 4).

Why would I–I species turnover be more influenced by geographical and environmental distance than M–I species turnover when the same overwater barriers and reductions in gene flow are likely to apply? First, islands may have shared geological histories unique from the mainland that would not be accounted for in the M–M model. For example, many islands in our study belong to island banks that formed large, connected landmasses during periods of low sea level (e.g. the Great Bahama Bank [34]). Resulting overland dispersal may have served to mix existing island assemblages, and increased gene flow between incipient species may have halted or reversed the speciation process and reset the speciation clock. Second, the relatively low average distance between islands (relative to M–I distances; electronic supplementary material, figure S2) and the likelihood that a non-random subset of capable dispersers [35] colonized the Caribbean archipelago from the mainland could have combined to make dispersal among islands more common than dispersal from the mainland to the islands, thus recovering the relationship between species turnover and geographical and environmental distance.

The closer match of terraranan I–I turnover to the mainland-predicted slope is perhaps counterintuitive, as amphibians are generally considered to be poor dispersers [36–38] and are expected to be more sensitive to salt water than reptiles. Thus, one might expect over-ocean dispersal limitation to have a greater effect on the terraranan I–I relationship. One possible explanation is that terraranans are also more limited by overland dispersal than anoles, perhaps because of susceptibility to desiccation. This is consistent with the smaller range sizes and higher rates of turnover among mainland terraranan assemblages compared with anoles. Thus, while terraranans may be poorer dispersers, the relative difference in overland and overwater dispersal may be lower for terraranans than anoles, leading to a smaller difference between M–M and I–I turnover relationships. In general, we predict that the greatest (or smallest) differences between mainland and island turnover will not occur in clades that are uniformly poor (or strong) dispersers, but rather in those clades that have low overwater dispersal ability relative to overland dispersal ability.

### (d) Caveats

An appreciable proportion of named anole and terraranan species are geographically restricted allopatric populations that are assumed but not known to be reproductively isolated from their sister species [16,17]. Because accurate estimates of species turnover depend on correct species assignment, taxonomic splitting of two populations that are quantifiably differentiated but not reproductively isolated could arbitrarily inflate estimates of species turnover, especially on islands where researchers may be more likely to delineate unique species. However, for anoles at least, a few recent studies have split mainland species [39], while several intraspecific genetic studies suggest that some island anoles may actually be under-split [40,41]. Furthermore, if the patterns we observed were due to an island taxonomic bias, then we would expect I–I turnover to be more elevated relative to the M–M relationship than M–I turnover; this was not the case. Last, closely related but allopatric populations and species of both clades are often substantially diverged at the molecular level [16,40], and anoles are often quite different in dewlap and body colour [42], while terraranans may often differ in their calls [43], altogether suggesting that differential splitting is not likely to have biased our results.

Our random selection of MSRs may have missed individual centres of mainland species endemism (e.g. mountain tops). Sampling these areas specifically could have led to a small additional number of high M–M turnover measures. However, our goal was to determine whether M–I and I–I turnover patterns differed from the general, representative M–M turnover patterns, rather than to focus on particular, possibly unrepresentative areas. Additional comparison of the turnover patterns of mainland centres of endemism to islands remains an interesting topic for future enquiry.

## 5. Conclusions

Our results show that faunal assembly is indeed unique on oceanic islands relative to mainland assemblages. For a given geographical and environmental distance between two localities, mainland–island and island–island assemblages have higher turnover on average than mainland–mainland assemblages, indicating that island biotas are, in fact, exceptionally unique. Higher turnover probably stems from the interaction of reduced dispersal, reduced gene flow, higher ecological opportunity and increased probability of speciation on islands generated by the inhospitable overwater barrier. Mainland–island turnover is higher than island–island turnover on average, possibly because the connectivity of islands on the same island bank during glacial high-stands serves to homogenize communities and collapse incipient species. More work is needed to understand whether islands influence other aspects of beta-diversity, such as phenotypic or phylogenetic turnover, in similar ways.
